# Understanding the Relationship Between Sarcopenia and Perioperative Outcomes Among Patients Undergoing Transjugular Intrahepatic Portosystemic Shunt: A Nationwide Analysis (2016–2022)

**DOI:** 10.14740/gr2162

**Published:** 2026-07-31

**Authors:** Mariam Alamgir, Bipneet Singh, Sahiljot Bhupal, Carol Singh, Aalam Sohal, Nilofar Najafian, Mohanad Al-Qaisi

**Affiliations:** a School of Medicine, St. George’s University, Grenada, West Indies; b Department of Hepatology, University of Kentucky, Lexington, KY, USA; c Department of Medicine, Dayanand Medical College and Hospital, Ludhiana, Punjab, India; d Division of Gastroenterology and Hepatology, Creighton University School of Medicine, Phoenix, AZ, USA

**Keywords:** Transjugular intrahepatic portosystemic shunt, Sarcopenia, Hepatic encephalopathy, Cirrhosis

## Abstract

**Background:**

Sarcopenia which is characterized by the loss of muscle mass, strength, and function is a common complication in patients with chronic liver disease and contributes to poor clinical outcomes. While transjugular intrahepatic portosystemic shunt (TIPS) has shown some improvement in muscle mass and body mass index in patients with sarcopenia, the impact of sarcopenia on post-TIPS morbidity and mortality has not been examined at a national level. This study aims to evaluate the effect of sarcopenia on outcomes in patients undergoing TIPS, to enhance management and improve clinical outcomes.

**Methods:**

We analyzed data from National Inpatient Database (NIS) database from 2016 to 2022, using ICD-10 codes to identify adult patients who underwent TIPS. Patients were classified into two groups, those with and without sarcopenia, based on ICD-10 sarcopenia codes. We compared the prevalence of perioperative adverse outcomes between the groups and used multivariate logistic and linear regression to assess the impact of sarcopenia on these outcomes.

**Results:**

A total of 19,610 patients who underwent TIPS were included in the study, with 3,510 (17.9%) diagnosed with sarcopenia. Patients with sarcopenia had a higher incidence of in-hospital mortality (11.8% vs. 7.7%, P < 0.001) and a greater overall complication rate (56.4% vs. 38.8%, P < 0.001), including gastrointestinal (10.3% vs. 3.6%, P < 0.001), cardiovascular (36.9% vs. 27.2%, P < 0.001), pulmonary (23.5% vs. 12.2%, P < 0.001), and infectious (14.1% vs. 5.5%, P < 0.001) complications. Patients with sarcopenia also had a significantly longer mean length of stay (14.7 vs. 7.0 days) and higher mean total charges ($280,818 vs. $159,272).

**Conclusion:**

This study demonstrates that patients with sarcopenia have worse outcomes and increased resource utilization in patients undergoing TIPS. Early identification and assessment of sarcopenia are crucial for identifying at-risk patients and guiding targeted interventions to improve recovery and reduce complications.

## Introduction

Sarcopenia is a general and progressive syndrome characterized by the loss of skeletal muscle mass and strength which can lead to physical limitations, poor quality of life, and increased mortality. While it predominantly affects the elderly, sarcopenia can also arise in younger individuals due to conditions such as malnutrition or chronic inflammatory diseases [1]. Previous studies estimate that sarcopenia affects 14% of individuals aged 65–70 years and rises to 53% among those above 80 years. The prevalence varies based on diagnostic definitions, with 5–13% of individuals aged 60–70 years and 11–50% of those over 80 years being affected [1]. As the global population ages, these numbers are expected to grow exponentially. An article by WHO predicts that the number of people aged 60 years and older is expected to increase from 1 billion in 2019 to 1.4 billion by 2030 and 2.1 billion by 2050, especially in developing countries. This demographic shift underscores the urgent need for effective strategies to address sarcopenia and its associated burdens [2]. In the United States, the direct healthcare costs attributable to sarcopenia were estimated at $18.5 billion in 2000, representing 1.5% of the total healthcare expenditures. Excess healthcare costs for individuals with sarcopenia amounted to $860 per man and $933 per woman annually. These costs underscore the financial burden of sarcopenia on healthcare systems and highlight the potential for significant economic benefits through preventive and management strategies [3].

Sarcopenia affects approximately 40–70% of patients with cirrhosis, contributing significantly to morbidity and mortality, and surpassing other complications such as variceal bleeding and hepatocellular carcinoma (HCC) in prevalence [3]. The unique metabolic derangements associated with cirrhosis, such as protein-energy malnutrition, systemic inflammation, and hormonal imbalances, exacerbate muscle wasting, highlighting the pressing need for targeted interventions [4]. Transjugular intrahepatic portosystemic shunt (TIPS) placement, a procedure primarily aimed at reducing portal hypertension and its complications, can lead to improvements in muscle mass and functionality [5, 6]. However, the interplay between sarcopenia and TIPS is complex; while TIPS can positively influence muscle recovery, sarcopenia itself has been identified as a risk factor for adverse post-TIPS outcomes, including hepatic encephalopathy (HE) and reduced survival [7, 8].

Given the bidirectional relationship between sarcopenia and TIPS outcomes, a deeper understanding of their interaction is crucial for patient selection and procedural strategies. Our study aims to explore the impact of sarcopenia on TIPS in patients with cirrhosis at a national level and seeks to aid clinical decision-making and improve patient outcomes. It also underscores the importance of integrating sarcopenia management into the broader care framework for cirrhotic patients undergoing TIPS.

## Materials and Methods

### Data source

Our analysis used National Inpatient Sample (NIS), which is a nationwide database maintained by the Healthcare Cost Utilization Project (HCUP) [9]. The NIS captures inpatient hospitalizations from a broad network of US hospitals and provides weighted estimates that reflect national patterns of care [9]. Every hospitalization is recorded as a separate, de-identified entry, allowing population-level analyses without the need for institutional review board (IRB) approval.

### Study population

**Figure 1:**
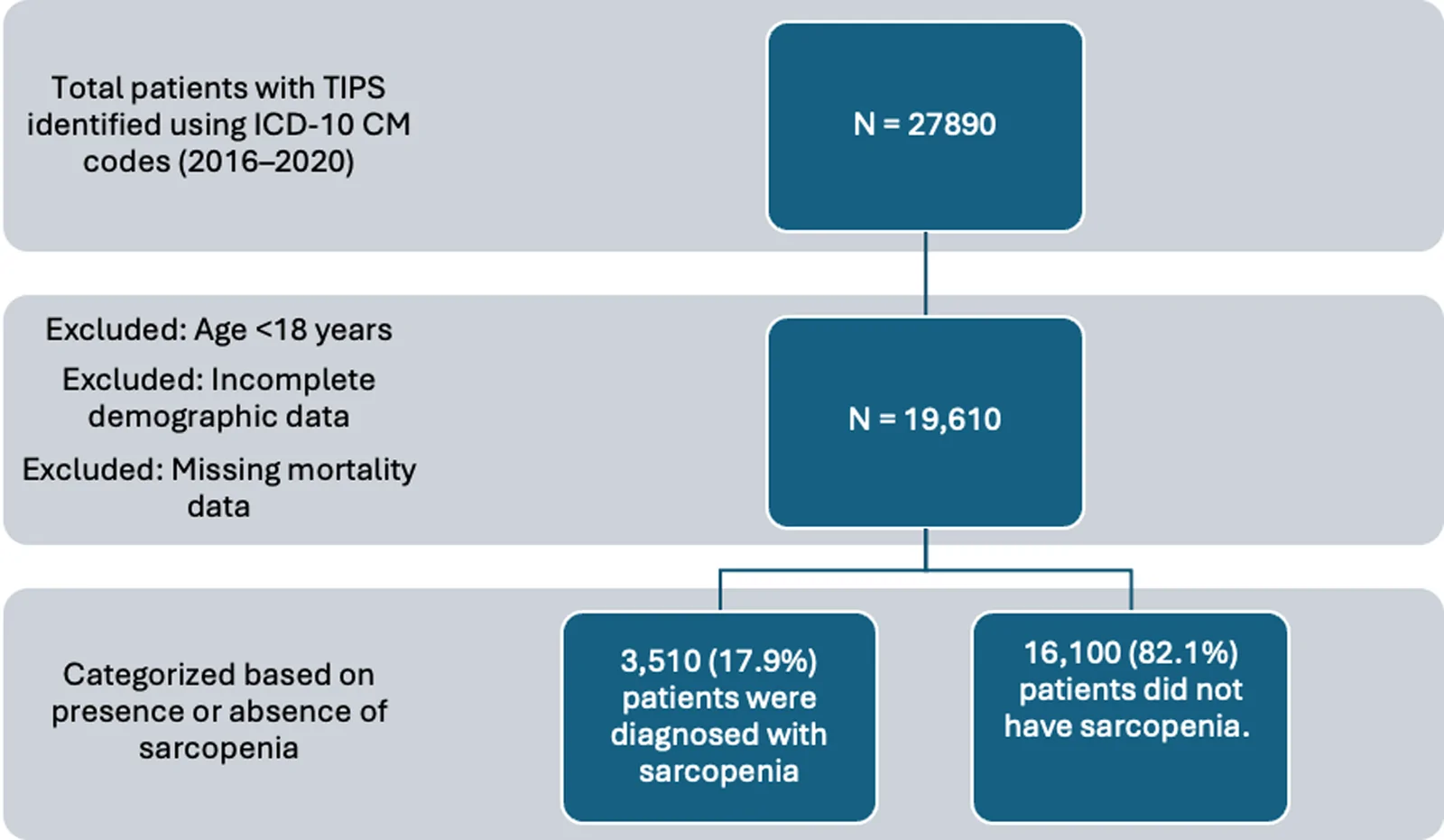
Inclusion criterion flowchart for patients undergoing TIPS included in the study. TIPS: transjugular intrahepatic portosystemic shunt.

Adult patients who underwent TIPS procedure between 2016 and 2020 were identified using the International Classification of Diseases, 10th Revision, Clinical Modification (ICD-10-CM) procedure codes (06183J4, 06184J4, 06183JY, and 06183DY). Individuals younger than 18 years of age and those missing demographic and mortality data were excluded. After applying these criteria, the final cohort consisted of 19,610 hospitalizations. Patients were then stratified into two groups based on the presence or absence of sarcopenia. Sarcopenia was identified using the ICD-10 code M62.84, supplemented by related diagnostic codes reflecting severe malnutrition (R63.4, R63.6, E43, E44.0, E46, E63.8, E63.9), cachexia (R64), muscle wasting (M62.5*), and adult failure-to-thrive (R62.7). This coding strategy has been used in previous database studies to improve capture of clinically relevant muscle loss [10–12]. Additionally, information was collected on comorbidities, including smoking, obesity, diabetes, coronary artery disease (CAD), hypertension (HTN), and obstructive sleep apnea (OSA) in our population. The inclusion process is detailed in Figure 1.

### Study variables

The study’s demographic variables (pre-specified by HCUP) included age, sex, race, primary insurance, and median household income quartile. Comorbidity burden was calculated from ICD-10 codes through the Charlson Comorbidity Index (CCI), which predicts mortality and hospital resource use in large cohorts [13].

Additional data were collected on underlying decompensations, procedures, disease etiologies, comorbid conditions, and procedure-related complications. Cirrhosis-related decompensations captured included ascites, spontaneous bacterial peritonitis (SBP), variceal hemorrhage, hepatorenal syndrome (HRS), HE, and HCC. In-hospital procedures included esophagogastroduodenoscopy (EGD), mechanical ventilation, and renal replacement therapy. With respect to etiology, alcohol-related liver disease (ALD), hepatitis B and C, metabolic dysfunction-associated steatotic liver disease (MASLD), Budd–Chiari syndrome, cholestatic liver disease, and autoimmune hepatitis were recorded. Other comorbidities include congestive heart failure (CHF), cardiac arrhythmias, renal failure, in-hospital mortality, gastrointestinal (GI) complications, cardiovascular complications, pulmonary complications, renal complications, infectious complications, neurologic complications, and postoperative shock. Patients who were discharged to a facility outside home, classified as non-home discharges, were collected. The ICD-10 codes used for complication identification are listed in Supplementary Material 1 (gr.elmerpub.com). All complications analyzed represent in-hospital post-TIPS events, as captured during the same hospitalization in which the TIPS procedure was performed. Pre-existing conditions were treated as comorbidities rather than outcomes.

### Statistical analysis

National estimates were generated using hospital-level discharge weights. Categorical variables were compared using the Chi-square test and continuous variables using independent sample *t-*tests. Analysis was done using multivariate logistic regression to evaluate the association between sarcopenia and post-TIPS complications while adjusting for confounding factors such as patient demographics, liver disease etiology, hepatic decompensations, inpatient procedures, comorbidities, and the CCI. Both unadjusted and adjusted odds ratios (ORs) with 95% confidence intervals (CIs) were reported. A P-value < 0.05 was considered statistically significant, and all analyses were performed in STATA 17.0 (StataCorp, College Station, TX).

## Results

### Patient demographics

Among the 19,610 hospitalizations meeting inclusion criteria, sarcopenia was identified in 3,510 patients (17.9%). Males accounted for 59.4% of the study population. The predominant age group was 45–65 years (54%), and most patients were White (70.8%) and in the lowest income quartile (31.8%). Full demographic data are presented in Table 1.

**Table 1:** Demographics of the Population Included in the Study

	Absence of sarcopenia, n (%)	Presence of sarcopenia, n (%)	P-value
Age categories			0.34
18–44	2,275 (14.1)	500 (14.3)	
45–65	9,150 (56.8)	1,895 (54.0)	
> 65	4,675 (29.0)	1,115 (31.8)	
Sex			0.05
Male	10,200 (63.4)	2,085 (59.4)	
Female	5,900 (36.6)	1,425 (40.6)	
Race			0.29
White	11,575 (71.9)	2,485 (70.8)	
African American	655 (4.1)	170 (4.8)	
Hispanic	2,800 (17.4)	585 (16.7)	
Asian/Pacific islander	295 (1.8)	95 (2.7)	
Native American	235 (1.5)	80 (2.3)	
Other	540 (3.4)	95 (2.7)	
Primary expected payer			0.43
Medicare	6,245 (38.8)	1,420 (40.5)	
Medicaid	3,950 (24.5)	940 (26.8)	
Private	4,555 (28.3)	870 (24.8)	
Uninsured	745 (4.6)	170 (4.8)	
Median household income			0.94
Lowest quartile	5,235 (32.5)	1,115 (31.8)	
Second quartile	4,190 (26.0)	895 (25.5)	
Third quartile	3,750 (23.3)	850 (24.2)	
Highest quartile	2,925 (18.2)	650 (18.5)	
Charlson Comorbidity Index			< 0.001
0	1,005 (6.2)	125 (3.6)	
1	590 (3.7)	40 (1.1)	
2	5,310 (33.0)	1,145 (32.6)	
3	9,195 (57.1)	2,200 (62.7)	

### Decompensation, etiology, and comorbidities

In our study, patients with sarcopenia demonstrated a substantially higher burden of decompensated cirrhosis. Compared with those without sarcopenia, they more frequently presented with ascites (80.2% vs. 65.3%), HRS (15% vs. 5.1%), SBP (8% vs. 2.5%), HE (14.8% vs. 7.1%), and renal failure (21.8% vs. 16.6%). Concomitant hepatitis B infection was also more common in the sarcopenia group (3.4% vs. 1.6%). During hospitalization, patients with sarcopenia required mechanical ventilation (27.8% vs. 20.5%, P < 0.001) and renal replacement therapy (7.8% vs. 3.4%, P < 0.001) more often than those without sarcopenia. A full summary of comorbidities and clinical characteristics is provided in Table 2.

**Table 2:** Decompensation, Procedures, and Comorbidities Stratified by the Presence of Sarcopenia

	Absence of sarcopenia, n (%)	Presence of sarcopenia, n (%)	P-value
Decompensation			
Ascites	10,505 (65.3)	2,815 (80.2)	< 0.001*
Hepatorenal syndrome	820 (5.1)	525 (15.0)	< 0.001*
Hepatocellular carcinoma	460 (2.9)	130 (3.7)	0.24
Spontaneous bacterial peritonitis	410 (2.5)	280 (8.0)	< 0.001*
Variceal bleeding	5,585 (34.7)	1,140 (32.5)	0.28
Hepatic encephalopathy	1,145 (7.1)	520 (14.8)	< 0.001*
Procedures			
Esophagogastroduodenoscopy	5,450 (33.9)	1,275 (36.3)	0.22
Blood transfusions	4,125 (25.6)	900 (25.6)	0.99
Mechanical ventilation	3,300 (20.5)	975 (27.8)	< 0.001*
Renal replacement therapy	540 (3.4)	275 (7.8)	< 0.001*
Etiology of liver disease			
Alcohol-related liver disease	7,900 (49.1)	1,820 (51.9)	0.19
Hepatitis C	2,695 (16.7)	545 (15.5)	0.41
Hepatitis B	255 (1.6)	120 (3.4)	0.001*
MASLD	4,960 (30.8)	875 (24.9)	0.002*
Budd–Chiari syndrome	190 (1.2)	40 (1.1)	0.93
Cholestatic disease	495 (3.1)	125 (3.6)	0.52
Autoimmune hepatitis	285 (1.8)	85 (2.4)	0.30
Comorbidities			
Congestive heart failure	1,435 (8.9)	425 (12.1)	0.006*
Cardiac arrhythmias	2,150 (13.4)	610 (17.4)	0.004*
Chronic renal failure	2,670 (16.6)	765 (21.8)	0.001*

*P < 0.05. MASLD: metabolic dysfunction-associated steatotic liver disease.

### Outcomes

**Figure 2:**
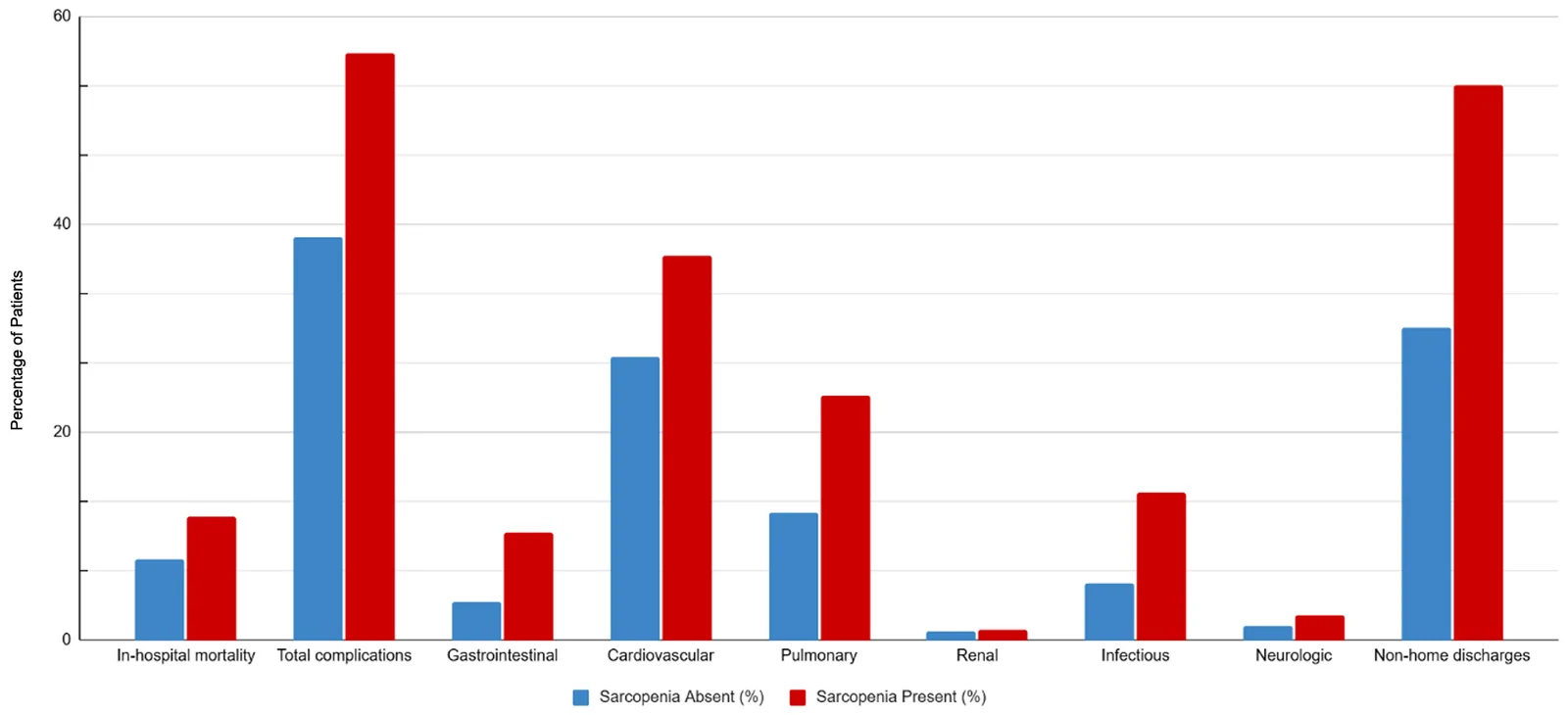
Distribution of outcomes in post-TIPS patients with and without sarcopenia. TIPS: transjugular intrahepatic portosystemic shunt.

Patients with sarcopenia who underwent TIPS were noted to have a higher incidence of total complications (56.4% vs. 38.8%, P < 0.001) compared with those without sarcopenia, as shown in Table 3. The burden of sarcopenia on clinical outcomes in TIPS patients is depicted in Figure 2 and Table 3.

**Table 3:** Outcomes Stratified by the Presence of Sarcopenia

Outcomes	Absence of sarcopenia, n (%)	Presence of sarcopenia, n (%)	P-value	Adjusted OR	95% CI	P-value
In-hospital mortality	1,245 (7.7)	415 (11.8)	< 0.001	0.95	0.67–1.36	0.789
Total complications	6,245 (38.8)	1,980 (56.4)	< 0.001	1.75	1.41–2.16	< 0.001
Gastrointestinal	585 (3.6)	360 (10.3)	< 0.001	2.5	1.83–3.43	0.038
Cardiovascular	4,385 (27.2)	1,295 (36.9)	< 0.001	1.32	1.01–1.71	< 0.001
Pulmonary	1,960 (12.2)	825 (23.5)	< 0.001	1.77	1.41–2.21	< 0.001
Renal	130 (0.8)	30 (0.9)	0.9004	1.13	0.44–2.91	0.795
Infectious	885 (5.5)	495 (14.1)	< 0.001	1.75	1.27–2.39	< 0.001
Neurologic	210 (1.3)	85 (2.4)	0.0268	1.54	0.81–2.92	0.188
Non-home discharges	4,850 (30.1)	1,875 (53.4)	< 0.001	2.2	1.77–2.73	< 0.001

CI: confidence interval; OR: odds ratio.

#### In-hospital mortality

The in-hospital mortality was higher among sarcopenic patients (11.8% vs. 7.7%, P < 0.001); however, after adjustment for comorbidities and illness severity, this difference was no longer statistically significant (adjusted odds ratio (aOR) 0.95, 95% CI 0.67–1.36, P = 0.789). The results of the multivariate logistic regression model are presented in Table 3.

#### GI outcomes

The incidence of adverse GI outcomes was higher among patients with sarcopenia (10.3%) than among those without sarcopenia (3.6%). On multivariate analysis, patients with sarcopenia had a significant difference in odds of developing GI complications (aOR 2.5, 95% CI 1.83–3.43, P = 0.038) when compared with patients without sarcopenia.

#### Cardiovascular outcomes

Cardiovascular complications occurred more frequently in patients with sarcopenia (36.9%) than in those without (27.2%). After multivariate adjustment, following adjustment for confounders, sarcopenia remained an independent predictor of cardiac complications (aOR 1.32, 95% CI 1.01–1.71, P < 0.001).

#### Pulmonary outcomes

Adverse pulmonary outcomes were noted in 23.5% of patients with sarcopenia compared with 12.2% of those without sarcopenia. On multivariate analysis, patients with sarcopenia had significantly higher odds of developing pulmonary complications (aOR 1.77, 95% CI 1.41–2.21, P < 0.001) when compared to patients without sarcopenia.

#### Infectious outcomes

Infectious complications were greater among patients with sarcopenia, occurring in 14.1% compared with only 5.5% in patients without sarcopenia. After adjusting for confounders, this association remained statistically significant on multivariate analysis, with sarcopenia independently conferring nearly twice the odds of infectious complications (aOR 1.75, 95% CI 1.27–2.39, P < 0.001).

#### Renal outcomes

Adverse renal outcomes were documented in 0.9% of patients with sarcopenia and 0.8% of patients without sarcopenia. On multivariate analysis, no statistically significant difference was observed between the two groups in the odds of developing renal complications after adjustment for confounding variables (aOR 1.13, 95% CI 0.44–2.91, P = 0.795).

#### Neurological outcomes

Adverse neurological outcomes were documented in 2.4% of patients with sarcopenia as compared with 1.3% of patients without sarcopenia. On multivariate analysis, no statistically significant difference was observed between the two groups in the odds of developing adverse neurological outcomes (aOR 1.54, 95% CI 0.81–2.92, P = 0.188).

#### Non-home discharges

The likelihood of non-home discharge was greater in patients with sarcopenia (53.4%) than among those without sarcopenia (30.1%). On multivariate analysis, after adjusting for confounding factors, patients with sarcopenia had significantly higher odds of undergoing non-home discharges (aOR 2.2, 95% CI 1.77–2.73, P < 0.001).

#### Resource utilization

##### 1) Length of stay

Patients with sarcopenia who underwent TIPS procedure demonstrated a substantially prolonged mean total length of stay compared with patients without sarcopenia (14.7 days, 95% CI 13.53–15.80 vs. 7.04 days, 95% CI 6.76–7.31). This pattern persisted when examining postprocedural length of stay specifically (9.49 days, 95% CI 8.58–10.40 vs. 4.64 days, 95% CI 4.43–4.85). On multivariate analysis, sarcopenia was independently associated with a significantly longer hospital stay after adjustment for confounding variables (adjusted coefficient 5.46, 95% CI 4.46–6.47, P < 0.001).

##### 2) Total hospitalization charges

Post-TIPS patients with sarcopenia had higher mean total hospitalization charges ($280,818, 95% CI 256,180.8–305,455.1) when compared with patients without sarcopenia ($159,271.7, 95% CI 152,858.2–165,685.3). On multivariate analysis, patients with sarcopenia had significantly higher total hospitalization charges after adjustment for confounding variables (aOR $79543.19, 95% CI 58,707.74–100,378.60, P < 0.001).

## Discussion

In this national cohort study of 19,610 patients undergoing TIPS, sarcopenia was present in one in six people undergoing the procedure. Sarcopenia in clinical practice is diagnosed using a combination of reduced muscle strength and objectively measured low muscle mass, with physical performance used to determine disease severity. It is likely that underdiagnosis due to ICD-10 coding may have led to capturing only severe cases of sarcopenia, leading to underestimation of the true burden. Our study noted sarcopenia to be associated with increased risk of worse outcomes and resource utilization. In our study, patients with sarcopenia had higher rate of decompensation such as ascites, HRS, SBP, and HE compared with those without sarcopenia. Previous clinical studies by Tapper et al and Nardelli et al have consistently shown that sarcopenia is an independent predictor of post-TIPS HE, increased hospitalization rates, and reduced transplant-free survival [14, 15]. In patients with cirrhosis, skeletal muscle serves as an alternative site for ammonia detoxification via glutamine synthesis; thus, sarcopenia compromises this compensatory mechanism, exacerbating hyperammonemia and predisposing to HE. The loss of muscle mass also impairs the muscle pump function, which contributes to poor venous return and worsening fluid retention, thereby exacerbating the development of ascites. Sarcopenia further reflects severe systemic inflammation with elevated proinflammatory cytokines (e.g., tumor necrosis factor-alpha, interleukin-6) and malnutrition, which accelerate circulatory dysfunction and renal hypoperfusion [14, 15].

In our study, sarcopenia was associated with higher total complications and non-home discharges. The most common complication in our study was cardiovascular complications. Sarcopenia may amplify vulnerability to post-TIPS hemodynamic stress. TIPS placement increases venous return and cardiac preload, which can unmask subclinical cirrhotic cardiomyopathy in patients with reduced physiological reserve, contributing to higher rates of cardiovascular and pulmonary complications. A study by Damluji et al has shown that patients with sarcopenia have a subclinical cardiac dysfunction, and Ansaripour et al demonstrated poor postsurgical cardiac outcomes in patients with sarcopenia [16, 17]. Our study also noted higher rates of infections among patients undergoing TIPS. This is consistent with a study by Zhang et al [18]. Sarcopenia impairs immune function through poor nutritional status and reduced production of immunomodulatory factors from muscle tissue, which may contribute to increased infection risk seen in our study.

Pulmonary complications were noted in one in four people undergoing the procedure. This may result from respiratory muscle weakness, impaired cough reflex, and increasing rates of mechanical ventilation, leading to an increased risk of pneumonia. GI complications, including pancreatitis, were more frequent in patients with sarcopenia. Chronic malnutrition and energy-protein deficits impair mucosal regeneration and compromise the intestinal barrier, increasing the risk of mucosal injury, ischemia, and bacterial translocation, which are key contributors to acute pancreatitis [19]. Furthermore, involvement of visceral smooth dysmotility due to intrinsic hormone imbalance leads to impaired GI motility, causing paralytic ileus [20]. Mucosal injuries, if severe, can even predispose organ perforation.

Renal and endocrine complications were infrequent following TIPS, irrespective of sarcopenia, with nearly identical rates between groups. These findings suggest that sarcopenia does not confer additional renal risk in the post-TIPS setting. The hemodynamic improvement in renal blood flow following TIPS likely overshadows the complications associated with physical debility. Similarly, neurologic complications, including infarction, edema, or hemorrhage, were slightly more frequent in sarcopenic patients compared with non-sarcopenic patients, but the difference did not reach statistical significance. A previous study by Kang et al showed that the poor nutritional status and physical debility were responsible for a worse prognosis, independent of the level of decompensation [21].

There was a higher incidence of in-hospital mortality compared with patients with sarcopenia than those without. After adjusting for confounding factors, the presence of sarcopenia was not associated with increased risk of in-hospital mortality suggesting that baseline severity among patients with sarcopenia is higher. Despite no apparent mortality differences, patients with sarcopenia had higher resource utilization including longer length of stay and higher total hospitalization charges. The increased cost could be partially attributable to the higher baseline liver disease severity as well as higher complication rates observed in patients with sarcopenia and TIPS, as demonstrated by our multivariate analysis.

### Limitations

This study has several limitations that should be considered when interpreting the findings. First, the analysis was based on the NIS and relies on ICD-10 coding which may underestimate the true prevalence of sarcopenia. The use of ICD-10 codes is also subject to misclassification. The approach used in our study to define sarcopenia is consistent with prior literature examining the association between sarcopenia and various acute and chronic GI conditions [10–12, 22, 23]. Second, the retrospective and observational nature of the study precludes establishing causal relationships between sarcopenia and TIPS-related complications, and residual confounding from unmeasured variables is possible despite multivariate adjustment. Third, data on TIPS indication and preprocedural interventions such as nutritional supplementation, physical therapy, or pharmacologic therapies are not available. Although the indication for TIPS was not available, we adjusted for possible indications of TIPS in our multivariate analysis, including variceal bleeding, HRS, ascites as well as Budd–Chiari syndrome. Due to the lack of granular data, we were unable to calculate Model for End-Stage Liver Disease scores. In order to account for this limitation, we used decompensations of liver disease as markers of disease severity. Additionally, information on long-term outcomes beyond hospitalization, including transplant-free survival, readmissions, and quality of life, was not captured. Despite these limitations, the study’s strength is the examination of a large cohort of patients undergoing TIPS.

### Conclusion

In conclusion, the analysis suggests the role of sarcopenia in post-TIPS complications. Incorporating sarcopenia assessment into pre-TIPS risk stratification may therefore offer an opportunity to improve outcomes. Prehabilitation strategies prior to elective TIPS placement and more vigilant monitoring of these patients after TIPS may help to reduce complications and prevent worse outcomes.

## Supplementary Material

Suppl 1 ICD-10 codes and associated system-wise complications used in the study.

## Data Availability

The authors declare that data supporting the findings of this study are available within the article.
