# Supplementary material for: Understanding the Relationship Between Sarcopenia and Perioperative Outcomes Among Patients Undergoing Transjugular Intrahepatic Portosystemic Shunt: A Nationwide Analysis (2016–2022)
Source: Gastroenterology Res. 2026 Jul 31;19(4):217–25. doi: 10.14740/gr2162 (PMC13568762; doi:10.14740/gr2162)
Supplement: Supplementary file 1 [file gr-19-04-217-s001.docx]

**Suppl 1.** ICD-10 codes and associated system-wise complications used in the study

| **Infectious complications** |  |
| --- | --- |
| A40 | Streptococcal sepsis |
| T814 | Infection following a procedure |
| R652 | Severe sepsis |
| T8653 | Shock due to anesthesia |
| **Neurologic complications** |  |
| G97 | Postprocedural neurologic complications |
| G46 | Vascular syndromes of cerebral infarction |
| G82 | Paraplegia and tetraplegia |
| G931 | Anoxic brain damage |
| G936 | Cerebral edema |
| I61 | Nontraumatic intracerebral hemorrhage |
| **Pulmonary complications** |  |
| J80 | Adult respiratory distress syndrome |
| J81 | Pulmonary edema |
| J90 | Pleural effusion, not classified |
| J91 | Pleural effusion, classified |
| J93 | Pneumothorax |
| J981 | Pulmonary collapse |
| R09 | Other signs/symptoms of circulatory and respiratory systems |
| J95 | Postprocedural pulmonary complications |
| T884 | Failed or difficult intubation |
| **Renal and endocrine complications** |  |
| E15 | Nondiabetic hypoglycemic coma |
| E272 | Addisonian crisis |
| E89 | Postprocedural endocrine complications |
| N139 | Obstructive and reflux uropathy, unspecified |
| N99 | Postprocedural genitourinary complications |
| **Cardiovascular complications** |  |
| I21 | Acute myocardial infarction |
| I126 | Pulmonary embolus |
| I46 | Cardiac arrest |
| I50 | Heart failure |
| R57 | Shock not elsewhere classified |
| I97 | Postprocedural CV complications |
| T801 | Vascular complications following infusion |
| T811 | Shock postprocedural |
| T817 | Vascular complications following procedure |
| **Gastrointestinal complications** |  |
| K560 | Paralytic ileus |
| K566 | Other intestinal obstructions |
| K569 | Ileus unspecified |
| K631 | Perforation of intestine nontraumatic |
| K638 | Other diseases intestine |
| K85 | Acute pancreatitis |
| K91 | Postprocedural GI complications |
| K223 | Perforation of esophagus |
| 3EO306 | Parenteral nutrition |
| 3E0336 | Parenteral nutrition |
| 3EO406 | Parenteral nutrition |
| 3E0436 | Parenteral nutrition |
| 3E0506 | Parenteral nutrition |
| 3E0536 | Parenteral nutrition |
| 3E0606 | Parenteral nutrition |
| 3E0636 | Parenteral nutrition |
| D7812 | Accidental puncture of spleen during other procedure |
| K9172 | Accidental puncture of digestive system organ during other procedure |
| K9162 | Intraoperative hemorrhage and hematoma of a digestive system organ or structure complicating other procedure |
| K91841 | Postprocedural hemorrhage of a digestive system organ or structure following other procedure |
